# Defining Breakfast in Dysphagia Care: Standards, Safety, and Nutrition

**DOI:** 10.7759/cureus.95787

**Published:** 2025-10-30

**Authors:** Carlo Pedrolli

**Affiliations:** 1 Nutrition Department, Santa Chiara Hospital, Azienda Provinciale per i Servizi Sanitari (APSS), Trento, ITA

**Keywords:** breakfast, community, dysphagia, hospital, nursing home

## Abstract

Breakfast research typically emphasizes the consequences of skipping the meal rather than the benefits of regular intake, and key terms and metrics remain inconsistently defined. Definitions differ across countries and study designs, which complicates the implementation of recommendations and can materially change nutritional assessments, as illustrated by comparisons of two practical definitions applied to a hospital menu. Evidence for breakfast in clinical populations with dysphagia is limited, despite the high risk of malnutrition and the routine dilution of foods that lowers nutritional density. Recent hospital nutrition guidance mentions breakfast only briefly and provides little direction on composition, timing within the morning window, or texture. We therefore advocate clear, uniform definitions of breakfast; dysphagia-specific breakfast guidance with explicit energy and macronutrient targets, with particular attention to protein; adoption of the International Dysphagia Diet Standardisation Initiative framework for food texture and liquid thickness; and a multidisciplinary approach involving dietitians, speech-language pathologists, and nurses to deliver safe swallowing alongside adequate nutrition.

## Editorial

Breakfast presents a paradox in the literature. Research has focused more on the effects of skipping breakfast, including its impact on metabolism [1], attention, weight, cognitive performance [2], and cardiovascular health, than on the benefits of regularly eating breakfast. A recent guideline from the International Breakfast Research Initiative offers evidence-based recommendations for a balanced breakfast for healthy individuals [3]. The main nutritional characteristics are summarized in the first row of Table 1 [3].

**Table 1:** Balanced breakfast guidance in healthy individuals and comparison of two hospital breakfast definitions (Santa Chiara Hospital, Trento) *Added sugar means the amount of "simple" carbohydrates (saccarose) permitted respectively in guideline, in breakfast simple and breakfast 6-11

Comparators	Medium daily intake (kcal)	Breakfast energy		Added sugar*	Total fat (%)	Saturated fat %	Protein (g)	Fiber (g)
		kcal	Percentage of daily kcal					
Guideline [3]	2000	300-500	20%	<10% of energy	20-30%	<10%	>10 g	>10 g
Breakfast simple	1878	264	14.0%	20 g	12.3%	Not reported	7.7g	0.6 g
Breakfast 6-11	1878	378	20.1%	20 g	18.4%	Not reported	11.2 g	0.6 g

Operational definitions vary substantially across studies and countries, which complicates the implementation of recommendations. In the six-country analyses we reviewed, participants in Canada, France, and the United States (US) self-defined breakfast; in the US, a minimum intake of >50 kcal was required; in Denmark and Spain, breakfast was captured through a specific questionnaire section; and in the United Kingdom, breakfast included “all items consumed between 6 am and 11 am.” From a practical standpoint, the time-window definition seems most useful for clinical settings. We applied two definitions, namely, “breakfast simple” (self-defined with the >50 kcal criterion) and “breakfast 6-11” (all items consumed between 6:00 and 11:00 am), to the breakfast offered at Santa Chiara Hospital, Trento (data on file, Hospital Santa Chiara, Trento, Italy; Table 1).

The difference was striking. Under “breakfast simple,” total calories were insufficient; protein and lipids were low; free sugar was excessive; and both approaches lacked fiber. The “breakfast 6-11” approach provided more calories, protein, and lipids, but the level of free sugar remained too high.

Evidence on breakfast in clinical contexts marked by dysphagia is notably sparse. This gap is surprising given that texture-modified foods and beverages are central to dysphagia care and are intended to mitigate key complications such as malnutrition, dehydration, and aspiration pneumonia. However, even recent hospital nutrition guidelines mention breakfast only in passing, emphasizing the interval between breakfast and dinner (more than 10 hours) without addressing breakfast composition, timing within the morning window, or texture considerations specific to dysphagia [4]. It remains unclear how often hospitalized or institutionalized patients with dysphagia receive an appropriate breakfast and whether its texture and nutritional profile align with therapeutic goals.

Meals hold particular importance for individuals with dysphagia across hospitals, nursing homes, and community settings. Clear, actionable guidance is needed on energy targets for breakfast, recommended food and beverage choices, and appropriate textures to support safe swallowing without compromising nutritional adequacy. As a practical path forward, we propose consistent and unambiguous definitions of breakfast for clinical and research use; development of breakfast guidelines tailored to dysphagia that specify caloric goals, macronutrient distribution (with particular attention to protein load), and recommended foods and beverages; adoption and routine use of standardized tools to measure food texture and beverage thickness, such as the International Dysphagia Diet Standardisation Initiative (IDDSI) framework [5]; and a firmly multidisciplinary approach. In this approach, dietitians, speech-language pathologists, and nurses collaborate to ensure that each patient receives the correct texture of foods and viscosity of beverages, along with a balanced, nutritious diet, using IDDSI as a common language to harmonize practice across settings.

## References

[1] Minari TP, Manzano CF, Yugar LB (2025). The effect of breakfast skipping and sleep disorders on glycemic control, cardiovascular risk, and weight loss in type 2 diabetes. Clin Nutr ESPEN.

[2] Cheng SH, Rebecca Yew LQ (2025). Breakfast skipping: influencing factors and its impact on cognitive function and academic performance among Malaysian University students. Percept Mot Skills.

[3] Gibney MJ, Barr SI, Bellisle F (2018). Towards an evidence-based recommendation for a balanced breakfast-a proposal from the International breakfast research initiative. Nutrients.

[4] Thibault R, Abbasoglu O, Ioannou E (2021). ESPEN guideline on hospital nutrition. Clin Nutr.

[5] Cichero JA, Lam P, Steele CM (2017). Development of international terminology and definitions for texture-modified foods and thickened fluids used in dysphagia management: the IDDSI framework. Dysphagia.

